# Cancer research provides a model for advancing clinical trials in dementia in the era of disease-modifying Alzheimer’s-type dementia therapies

**DOI:** 10.1186/s13195-024-01532-6

**Published:** 2024-08-21

**Authors:** Gregory A. Jicha, Thomas C. Tucker, Susanne M. Arnold, Peter T. Nelson

**Affiliations:** 1 Department of Neurology, University of Kentucky, Lexington, KY USA; 2 Department of Internal Medicine, University of Kentucky, Lexington, KY USA; 3 Department of Pathology and Laboratory Medicine, University of Kentucky, Rm 575 Lee Todd Bldg, 789 S. Limestone Ave, Lexington, KY 40536 USA; 4 Sanders-Brown Center On Aging, University of Kentucky, Lexington, KY USA; 5 College of Public Health, University of Kentucky, Lexington, KY USA; 6 Markey Cancer Center, University of Kentucky, Lexington, KY USA

**Keywords:** Lecanemab, Donanemab, Epidemiology, Immunotherapy, Alzheimer’s disease, Neuropathology, Side effects, Polling, Non-amnestic, Neuropsychiatric

## Abstract

Dementia and cancer are multifactorial, widely-feared, age-associated clinical syndromes that are increasing in prevalence. There have been major breakthroughs in clinical cancer research leading to some effective treatments, whereas the field of dementia has achieved comparatively limited success in clinical research. The lessons of cancer research may help those in the dementia research field in confronting some of the dilemmas faced when the clinical care regimen is not entirely safe or efficacious. Cancer clinical trials have assumed that untreated individuals with cancer are at high risk for morbidity and mortality after primary diagnoses. Thus, patients deserve a choice of clinical interventions, either standard of care or experimental, even if the benefits are not certain and the therapy’s side effects are potentially severe. The prognosis for many individuals at risk for dementia carries a correspondingly high level of risk for both mortality and severe morbidity, particularly if one focuses on “health-span” rather than lifespan. Caregivers and patients can be strongly impacted by dementia and the many troubling associated symptoms that often go well beyond amnesia. Polls, surveys, and a literature on “dementia worry” strongly underscore that the public fears dementia. While there are institutional and industry hurdles that complicate enrollment in randomized trials, the gravity of the future morbidity and mortality inherent in a dementia diagnosis may require reconsideration of the current protective stance that limits the freedom of at-risk individuals (either symptomatic or asymptomatic) to participate and potentially benefit from ongoing clinical research. There is also evidence from both cancer and dementia research that individuals enrolled in the placebo arms of clinical trials have unexpectedly good outcomes, indicating that participation in clinical trial can have medical benefits to enrollees. To highlight aspects of cancer clinical research that may inform present and future dementia clinical research, this review highlights three main themes: the risk of side effects should be weighed against the often dire consequences of non-treatment; the desirability of long-term incremental (rather than “magic bullet”) clinical advances; and, the eventual importance of combination therapies, reflecting that the dementia clinical syndrome has many underlying biological pathways.

## Background

Many clinical trials aimed at curing or preventing dementia have been unsuccessful to date and there has been reluctance from the general population to participate in dementia-oriented clinical trials [1, 2]. The partial success of Aβ immunotherapies, and the controversy surrounding their use, only increases the urgency for data-driven discussions in the field [3]. Here we focus on several heuristic concepts related to the field of dementia research using cancer research as source of possible future directions.

**Fig. 1:**
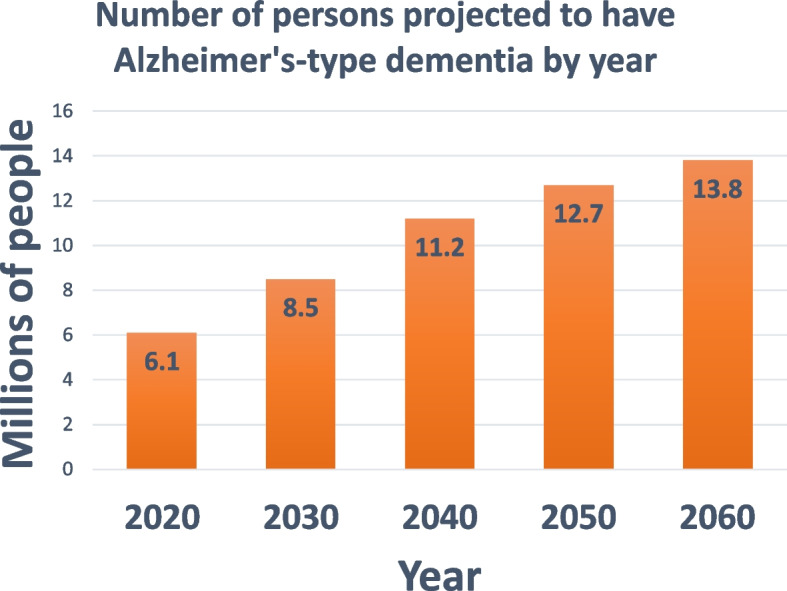
A large and increasing public health problem. The burden of Alzheimer’s-type dementia is predicted to increase over upcoming decades with aging of the population. Source: *Alzheimer’s Facts and Figs.* 2023 [46]

Dementia (also termed “major neurocognitive disorder”) is a clinical syndrome defined by memory loss and progressive impairment that compromises a person’s abilities to perform activities of daily living [4]. The clinical course of neurodegenerative dementias lead to increasing disability and death. Dementia is an extremely large public health problem that is positioned to become even worse as population demographics shift toward larger numbers of individuals surviving past 80 years of age (Fig. 1). The financial burden of dementia care is enormous (estimated at $335 billion/yr in the United States alone) [5], and the stress can be extreme for patients and caregivers alike.

The *sine qua non* for clinical trials is ethical responsibility, and the broader aspiration is to improve public health. Beyond those unshakable tenets, an underlying supposition for the present review article is that, to improve on the present model of clinical dementia research, it is logical to gather information about a successful parallel clinical research model. There is now a large corpus of relevant research findings from both the cancer and dementia research fields.

As in cancer research, dementia research stakeholders are increasingly acknowledging the complexity of the underlying biology of age-related cognitive impairment. Neither cancer nor dementia is a distinct disease – each is a clinical syndrome, with many complex pathways that require correspondingly complex diagnostic and therapeutic considerations if we are to succeed in our efforts to improve clinical research. Both dementia and cancer have populations defined not only by the disease, but by a continuum of phenomena that change as the disease(s) advance from early stages to late/terminal stages, offering different populations for research evaluation, and different levels of risks that these populations may be willing to accept in clinical trials.

As such, a deliberate approach that accounts for factors in common with cancer research may lead to improved standard of care paradigms for dementia syndromes and the diseases that are responsible for such syndromes. Here we emphasize three main themes: the acceptance of risk of side effects under some circumstances for a dreadful disease without curative standard treatments; the advantageous facets of incremental (rather than “magic bullet”) clinical advances; and, the eventual importance of combination therapies, reflecting that the dementia clinical syndrome, analogous to cancer, has many underlying biological pathways (main points described in Table 1).

**Table 1:** Summary points

Shared or parallel features of cancer and dementia	Why this is important
Cancer and dementia are both widely feared age-related diseases	There is a population of individuals who would welcome the opportunity to participate in clinical trials, and it is an ethical goal to better serve them
At-risk individuals face relatively rapid decrease in lifespan (cancer) or healthspan (dementia)	Persons with incipient pathology have high risk for medical problem so risk/reward calculation may favor intervention in near term
Heterogeneous and complex etiologies underlying the clinical syndrome	"No silver bullet" will cure all patients, yet subsets of individuals may be targetable and therapeutic combinations may work best
Patients enrolled in clinical trials receive optimal care	Ethical considerations may favor involvement in clinical trial even if patient is in placebo arm of study
Emphasis on giving patients choices	Since the most targetable disease is the earliest stages, at-risk individuals may be given education and appropriate consenting process

## Main text

### Choosing the level of risk to accept, factoring in the risks of non-participation in clinical research

A fundamental concern related to any clinical trial is the risk/benefit calculation, which includes consideration of the disease’s “natural history”. Perhaps the most relevant basic question is: what are the risks of doing nothing? The dementia clinical syndrome is lethal and often highly stressful to patients and caretakers. At least three common features of the dementia diagnosis seem noteworthy: 1. The trajectory of memory loss and “global” cognitive impairment; 2. Troubling dementia-linked symptoms other than amnesia; and, 3. The degree to which the lay public fears dementia symptoms and is willing to accept therapeutic risk of clinical trials. All of these factors may help inform the calculation about whether patients wish for – and should be more actively provided with – more choices in terms of their participation in clinical trials that may ultimately lead to improved care models.

Cancer therapeutic trials have used a similar “risk versus curative potential” to define the appropriateness of offering trials to patients with different stages of cancer. The oftentimes grim implications of a cancer diagnosis are a main factor leading to approval of anti-neoplastic clinical trials by oversight bodies, despite significant medical risks associated with some experimental therapeutic strategies. The clinical course of cancers can range from months to years. By comparison, the cognitive symptoms of dementia are usually slower than cancer, occurring over ~ 4–8 years after diagnosis, depending on disease subtypes, the patient population, and the criteria used to diagnose them. Yet in recent years, the assessment of clinical outcomes has shifted from a focus on life-span (number of years of life remaining) toward the concept of “healthspan”, which includes an assessment of the quality of life [6]. This is an important concept because, due to medical technologies and practices, many individuals are extending longevity but experiencing severe incapacity, due to dementia-related diseases. The fact that so many of demented subjects’ final years are spent in profoundly debilitated states make the concept of “survival” a less relevant concept for consideration in relation to dementia compared to many forms of cancer which typically demonstrate a swifter terminal decline.

As described in the Alzheimer’s Facts and Figures 2021 [5]:

“A person who lives from age 70 to age 80 with Alzheimer’s dementia will spend an average of 40% of this time in the severe stage [7]. Much of this time will be spent in a nursing home. At age 80, approximately 75% of people with Alzheimer’s dementia live in a nursing home compared with only 4% of the general population age 80 [7]. In all, an estimated two-thirds of those who die of dementia do so in nursing homes, compared with 20% of people with cancer and 28% of people dying from all other conditions” [8].

In addition to increased survival, a preventative treatment would avert or attenuate this profoundly debilitated stage for many–entailing not only a longer life, but quantumly lower morbidity.

**Fig. 2:**
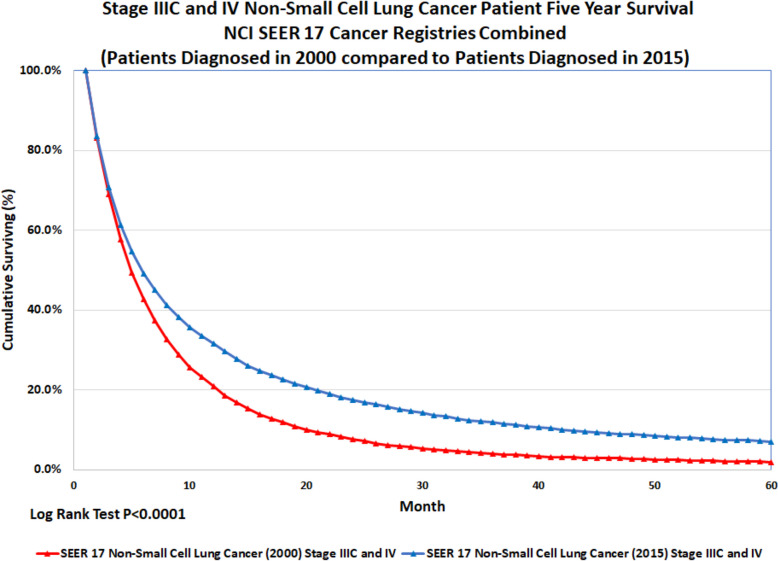
Bending the survival curve. Lung cancer remains the leading cause of cancer deaths. However, this common medical condition also represents an example of how aggressive clinical trials have significantly improved the survival of cancer patients with advanced disease. Shown here is a chart that shows five year observed survival for the NCI SEER 17 Cancer registries late stage non-small cell lung cancer patients diagnosed in 2015 compared to those diagnosed 15 years earlier (2000). Source: *NCI SEER Cancer Data Set* (Ref [9])

Yet lifespan itself is important. Although subtypes of cancer remain challenges in the clinical setting, oncologists have been successful at bending the survival curves. The chart in Fig. 2 shows data from National Cancer Institute (NCI) Surveillance, Epidemiology, and End Results (SEER) Program 17 data set [9] depicting survival curves (comparing those diagnosed in 2000 with others diagnosed in 2015) for non-small cell carcinoma of the lung, which is responsible for the deaths of more than 130,000 individuals each year in the United States. Several observations are notable about this prevalent and aggressive class of cancer: 1. More than 1/4^th^ of individuals who were diagnosed with non-small cell lung carcinoma were still alive 1 year after diagnosis; 2. ~ 5–10% of people were alive 5 years after diagnosis; and 3. There has been incremental improvement in clinical outcomes over the time-interval from 2000–2015. These data illustrate that the majority of patients can expect imminent mortality with stage IV lung cancer and may help to explain why cancer patients are incentivized to participate in clinical trials that may extend their health-span and lifespan.

How about the clinical syndrome of dementia? The popular stereotype of dementia relates primarily to a loss of memory, i.e. amnesia. Although amnesia is a cardinal clinical feature of dementia, there are many additional prevalent signs and symptoms that are often predominant and/or comorbid in persons with dementia. An assessment of clinical trial participation risks and potential benefits should factor in these harmful clinical comorbidities that occur in the course of the disease, although mortality is the long-term endpoint of dementia.

The prototypic patient with dementia was Mrs. Auguste Deter, a patient of Dr. Alois Alzheimer who was the index case of the disease that became known as Alzheimer’s disease [10]. Mrs. Deter was autopsied in 1905 and histologic examination of her brain revealed the silver-impregnable plaques and tangles that are still the pathological hallmarks of the eponymous disease. Although her clinical course had evolved to what is currently described as dementia, paranoid delusions were the presenting symptoms that initially troubled Mrs. Deter and vexed her family, and later she experienced sleep problems, aggressiveness, uncontrolled weeping, and severe apathy [10]. To the present day, dementia patients continue to be distressed by non-amnestic symptoms including delusions, hallucinations, agitation, irritability, depression, anxiety, disinhibition, motor dysfunction, apathy, eating disorders and/or sleep disturbance [11, 12].

**Fig. 3:**
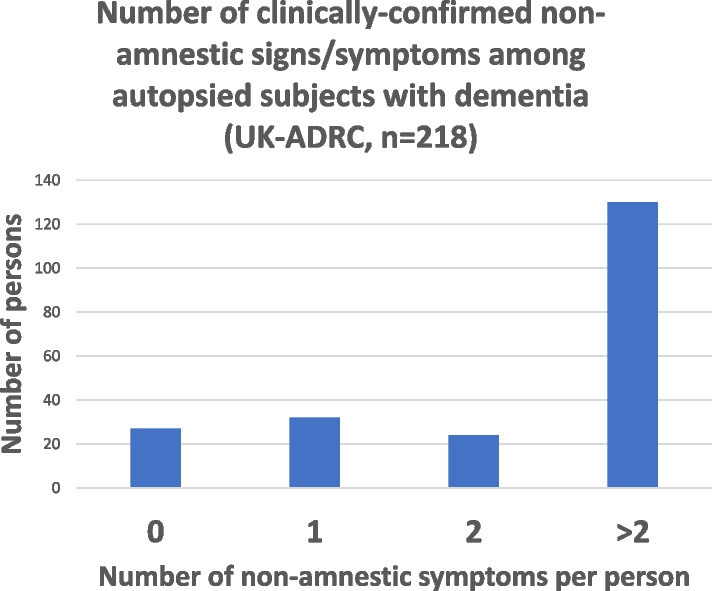
Alzheimer’s-type dementia is not just a memory disorder. Non-amnestic symptoms of dementia include agitation, anxiety, depression, sleep disorders, language problems, executive dysfunction, autonomic problems, motor problems, and others. See Table 2; these represent results from the University of Kentucky Alzheimer’s Disease Center cohort [13], a community-based autopsy cohort following research participants longitudinally from normal cognition. The severity and multiplicity of symptoms in dementia is an important consideration when contemplating the use (and the consequences of withholding) of clinical remedies

To illustrate the scale of the problem of non-amnestic symptoms in dementia, Table 2 and Fig. 3 show data derived from autopsied research volunteers (after having a clinical history of dementia) who were followed in the community-based cohort at the University of Kentucky AD Research Center (ADRC) [13]. In this sample, over 70% of evaluated demented subjects had at least one, and almost ½ of the subjects had more than one of these highly stressful symptoms, in addition to profound amnesia, often occurring long before death. All these are among the harms that are associated with non-treatment of persons at risk for dementia.

**Table 2:** Non-amnestic clinical features among subjects with dementia (*n* = 218) followed at UK-ADRC

Non-amnestic sign/symptom	% of subjects
Apathy	50.7
Depression	42.3
Anxiety	39.4
Motor dysfunction	39.4
Irritability	39.0
Agitation	36.2
Night/sleep problems	36.2
Disinhibition	35.7
Delusions	29.1
Hallucinations	21.6
Appetite dysfunction	20.2
Elation	2.8

Further, the impact of a dementia clinical syndrome is not limited to the patients themselves. The “risk of dementia” – the costs of only providing standard-of-care, outside of clinical trials – should acknowledge the extreme burden faced by caregivers, loved ones and society in general. Some of the challenges for caregivers include emotional trauma (often triggering depression and other physiologic sequelae including chronic diseases and even death), lost wages, and other adverse consequences [5, 14]. The travails of dementia care givers are not dispositive factors in many clinical trials for dementia because the focus on the patient is a primary consideration in most studies. Yet from a public health perspective, reductions in caregiver burden is a societal challenge that is important to consider when evaluating the risk/benefit of including experimental clinical interventions to standard-of-care treatment approaches.

Although epidemiologic studies indicate that dementia has a very large adverse impact on public health [5], it does not necessarily follow that the lay public has a commensurate fear of an imminent development of cognitive decline and functional compromise inherent in a diagnosis of dementia. This is a pertinent consideration because clinical trials are only warranted if at-risk individuals are potentially willing participants in clinical trials. Although there is no perfect metric to indicate the amount of concern that is attached to a clinical diagnosis among the lay public, there are numerous indications that the public is aware of dementia as a grave public health problem.

One pertinent concept is “dementia worry”, defined as “an emotional response to the perceived threat of developing dementia, independent of chronological age and cognitive status” [15]. From scholarship on dementia worry, there is good evidence that people are aware of, and anxious about, the prospects of receiving the diagnosis of dementia in their lifetime. As one may expect, measured dementia worry is increased in people with personal experience with a demented loved one and/or known genetic risk for dementia, as well as individuals with symptoms they interpret to be early warning signs [15, 16]. As such, dementia worry is linked to a willingness to be screened and tested for objective indications of risk [15]. However, engagement in clinical trials for the prevention of dementia remains problematic and delayed or ineffective recruitment into clinical research in dementia stands as a major barrier to success for clinical trial endeavors.

There also have been numerous public polls that queried individuals about the age-related diseases that they most fear. These studies are outside of the peer-reviewed academic literature, but often have employed scientific methodology. It is challenging to interpret these polls comprehensively, but we highlight a sampling of polling (and some surveys') results in Table 3. Note that in polls conducted soon after the COVID-19 pandemic began, the fear of dementia remains still above that of COVID-19. The non-peer reviewed nature of public polling should be kept in mind, but these data provide evidence indicating that the lay public strongly fears dementia. From these polls and the studies on dementia worry, it is a credible corresponding hypothesis that many lay persons would be willing partners in a stronger effort to support clinical trials in dementia. Further understanding is required about the barriers to engagement in clinical trials for dementia prevention and treatment.

**Table 3:** Public polling results related to lay persons’ fears of Alzheimer’s and dementia

Poll/Survey	Sample	Year	Notes	URL
Saga Survey, U.K	9,116 50 + yr olds	2016	Dementia most feared condition by 43%, cancer most feared by 30%	https://newsroom.saga.co.uk/news/dementia-more-feared-than-cancer-new-saga-survey-reveals
YouGov, U.S	1,294 18 + yr olds	2021	49% are concerned about Alzheimer's disease	https://today.yougov.com/topics/health/articles-reports/2020/01/08/alzheimers-dementia-poll-survey
YouGov, U.K	2,049 18 + yr olds	2021	Dementia most feared condition by 38%, cancer most feared by 26%	https://recognitionhealth.com/yougov-survey/
Medicare Advantage poll, U.S	1,221 18 + yr olds	2021	84% feared cancer, 79% feared Alzheimer's disease, 67% feared Covid-19	https://www.medicareadvantage.com/news/most-feared-heath-conditions-report
(Academic survey)	355 adults at Austrialian outpatient clinic	2023	Cancer most feared at 34%, dementia second-most at 29%	PMID 37173717
Forbes Health Survey	2,000 U.S. adults	2024	Cognitive decline/ Dementia among top health-related fears at 44%	https://www.forbes.com/health/medicare/fear-of-aging-survey/

### Clinical trials and therapies for asymptomatic/occult disease

Using biofluid- and neuroimaging-based biomarkers, clinicians are increasingly able to detect evidence of pathophysiologic disease (and corresponding increased risk), even in neurologically normal individuals. As in the case of cancer, it is these pathologic mechanisms and lesions, rather than the symptoms, that are now considered the specific indications of the presence or absence of the disease in a biologic sense [17, 18]. However, there may be an aversion to treating neurologically unimpaired persons with a therapeutic strategy that is expensive and/or has side effects according to the non-maleficence maxim of “*primum non nocere*” (first, do no harm). Here we have another area where cancer research provides parallels and insights, but also important contrasts.

Cancer clinical trials for occult disease or prevention of recurrence in asymptomatic individuals are very common, and recruitment into those trials is robust and not problematic, in general. Trials of adjuvant therapy after surgery in asymptomatic individuals have been performed in a multitude of cancers, representing a common treatment paradigm [19]. But in contrast to AD, there are many therapeutic anti-cancer agents that have demonstrated efficacy in later stage disease, which can be selected for prevention of disease recurrence with confidence that they are known to kill cancer cells. This is the classical trial development paradigm of cancer—initially assess drugs in late stage disease and then design subsequent studies to assess the same therapies in earlier stages of disease and/or to prevent recurrences. Dementia research is still waiting for drugs that are effective in late stage disease (reverses symptoms, rather than delaying symptom onset) in order to develop subsequent trials in prevention of early stage disease. Such drugs may unfortunately be practically impossible because it may be futile to target neurodegenerative diseases after the complex brain connectome is destroyed and/or the pathogenetic process becomes auto-propagating [20]. This is a fundamental difference in approach—the AD/ADRD research discipline may need to start with prevention, whereas the cancer research discipline could focus initially on treatment after disease manifestation, and moved backwards to prevention.

Despite crucial differences in the cancer-research and dementia-research fields, we can still learn from how cancer researchers have addressed asymptomatic patients with cancer diagnoses. For example, an important asset that is used nearly universally in cancer clinical care but more rarely in dementia research is the data-driven education of patients with probabilistic survival and healthspan statistics. Consider how virtually any type of cancer diagnosis will be conveyed to the patients along with probabilistic expectation of 5-, 10-, and/or 20-year survival, whether or not the patient is symptomatic. This resource could also be welcome for both symptomatic and asymptomatic patients with biomarker-diagnosed AD/ADRD risk: what are their probabilistic risks, and how would the therapeutic strategy perhaps alter that risk? The practical and ethical issues of patient care should be viewed through this lens as we seek to give patients informed choices about their best treatment (or watchful-waiting) strategies.

### Accepting incremental clinical benefits, versus holding out for the “magic bullet”

Practical considerations have catalyzed a relatively successful approach in cancer research that incorporates increased clinical trial participation, breadth of investigations, “shots on goal”, and, “take the win when you can” as paradigms that occur in the context of a medically ethical approach (see for example Refs [21–24]). A compassionate, data-driven, and rigorous set of approaches to treat chronic disease states like cancer or dementia do not aspire (in the short term) to necessarily cure most, much less all, treated individuals: it is quantumly better for a drug to work on 5% of cases than 0%. As such, incremental clinical gains provide medical improvements for individuals who are helped, and also constitutes a critical starting-point to achieve greater – more effective, safer, and perhaps more cost-effective – progress in the future.

Nor is it necessary or implicit that any given anti-cancer therapeutic strategy will prove effective for any given cancer patient. This is partly because of the aggressiveness of many cancers, but also because there are assumed to be idiosyncratic interactions between a given person’s biologic processes and a therapeutic intervention. To address this issue, the field of cancer research has actively grappled with the complexity of different cancers’ molecular phenotypes, which has led to dramatic breakthroughs in targeted therapeutics and therapeutic efficacy in recent years [25].

To accommodate the patients’ desires for therapeutic intervention, the cancer field has been amenable to a wide variety of potentially harmful and quite expensive therapeutic strategies including surgical, radiation, immunotherapies, and other modalities. There also are a number of extremely costly anti-cancer therapies that are FDA-approved despite a somewhat modest impact on survival or morbidity. For example, glioblastoma, among the most aggressive and common subtypes of brain cancer, may be treated with a surgically implanted (Carmustine) chemotherapy-secreting material called Gliadel wafers [26]. The surgical implantation of FDA-approved Gliadel wafers costs over $50,000 and, on average, improves survival by less than two months [27]. Without debating the merits or demerits of any given therapeutic approach, we emphasize that the overall paradigm adopted by the cancer researcher community decades ago has been effective at extending the survival of gliomas [28] and many other types of cancers.

An over-arching question is: who exactly benefits from clinical trials? There is a relevant scientific literature comparing outcomes of patients who participate in clinical trials – but who only receive a placebo – with those who do not participate in clinical trials at all. This phenomenon has been termed the “trial effect”, and has been suggested to indicate (in the cancer research literature) that randomized clinical trials have an intrinsic benefit for recruited subjects [29, 30]. Analogous results have also been obtained in dementia clinical trials [31]. These data should be interpreted keeping in mind that patients who participate in clinical trials trend toward high socioeconomic status, and always “opt in”, and therefore represent a highly self-selected population. However, at the very least it underscores the importance of patient education and indicates the utility of having the type of medical attention that is seen in clinical trials – whatever the disease target may be. Whereas more patients in clinical trials would be desirable in some ways, it could still be unethical to recruit participants into clinical research if the proposed studies were unhelpful and/or involve injurious exposures, a common focus of Institutional Review Boards (IRBs) across the country. Even wasting a patient’s time, letting the patient know their risk category inappropriately, or providing false hope could be injurious. These points of concern must be addressed, and each trial must be evaluated cautiously on a case-by-case basis by clinicians and IRBs as is the standard for research currently.

### Multiple dementia-driving comorbidities may require multiple therapies

While dementia, like cancer, is widely dreaded, it is also abundantly clear that neither dementia nor cancer are single diseases. They are, instead, complex syndromes with many underlying contributory pathogenetic biologic pathways. Addressing the complexity of cancer-driving mechanisms has enabled some of the cancer field’s success stories as cancer researchers have accomplished more specific, tailored, and less toxic therapeutic strategies. We here describe some of the relevant studies in the areas of clinical-pathologic correlation, which provide added context relevant to dementia clinical trials.

In the field of oncology, rather than an expectation of a primary diagnosis that is restricted to “yes-cancer versus no-cancer”, there is a paradigm of tailoring therapies to a complex and disease subtype-specific biological signature. More specifically, there is a focus on the genetics and molecular biomarkers that differentiate one person’s cancer subtype from another’s. This process has led to a model of clinical care where the pathological diagnosis is a critical pivot-point in a given cancer patient’s medical management.

By contrast, the dementia field has tended to dichotomize based on both clinical (“yes-dementia, no-dementia”) and pathological (“AD versus not-AD”) model that masks the true complexity in both pathogenetic processes and their clinical phenotypes. This is an unfortunate tendency since so many aged individuals have gradations of neurological impairment and also complex neuropathologies and/or underlying genotypes. The pathologic phenotypes that we hypothesize to be substrates of dementia in aging are also heterogeneous: there is a wide spectrum of disease pathologies represented in human populations, and also complex mixtures of pathologies that commonly affect specific individuals. In community-based autopsy cohorts, the presence of “pure” pathologic subtypes are relatively unusual [32, 33]. This complexity is analogous to that seen in the field of oncology but has yet to be embraced broadly in the dementia research culture as a clinically-relevant concept.

Most dementia researchers are aware of some of the dementia-related neuropathologies, for example: Aβ and tau in amyloid plaques and neurofibrillary tangles of Alzheimer’s disease neuropathologic changes (ADNC) [34]; α-Synuclein in Lewy body diseases (LBDs) [35, 36]; and, TDP-43 proteinopathy in frontotemporal lobar degeneration (FTLD-TDP) [37] and in limbic-predominant age-related TDP-43 encephalopathy neuropathologic changes (LATE-NC) [38]. Vascular cognitive impairment and dementia (VCID) is a separate category of diseases that encompass the different ways that cerebrovascular disease can contribute to the dementia clinical syndrome [39] (for example, recent studies have indicated that small vessel diseases including brain arteriolosclerosis can have a substantial contribution to dementia [40]).

**Fig. 4:**
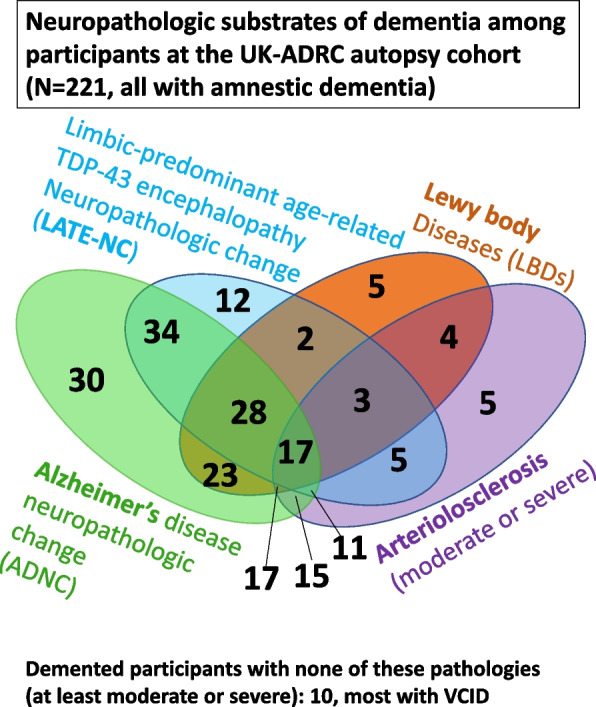
Dementia is not just Aβ amyloid plaques and tau neurofibrillary tangles. The neuropathologic substrates of “Alzheimer’s-type” dementia (i.e., changes that are observed in dementia brains at autopsy) are far more complex than the classic pathognomic features of Alzheimer’s disease, namely amyloid plaques and neurofibrillary tangles. Other prevalent conditions include limbic-predominant age-related TDP-43 encephalopathy (LATE), Lewy body diseases (LBDs), and vascular pathologies including arteriolosclerosis. Shown here are the results from the results from the University of Kentucky Alzheimer’s Disease Center autopsy cohort [13]. Note that in this sample, as in others, only a minority of “Alzheimer’s-type dementia” brains show “pure” plaques and tangles at autopsy– 30 out of 221 cases, or < 14%. Instead, most brains show a mixture of different pathologies. In the future, it may be possible to diagnose patient’s individual risk profile and target the person-specific relevant pathogenic pathways, perhaps using multiple remedies, as is done with cancer therapeutics and adjuvants

The combinations of pathologies are numerous and demonstrate that a “silver bullet”-type dementia therapy will be challenging if not impossible. Shown in Fig. 4 are the pathologies observed in individuals with documented dementia at the UK-ADRC that came to autopsy [13, 41]. Note that < 14% (30/221) of the demented subjects in this cohort have “pure” ADNC, whereas, for example, more have the combination of ADNC, LATE-NC, *and* LBDs.

These observations are directly relevant to dementia clinical trials. It is a basic hypothesis that the strategies of therapeutic interventions address specifically the disease(s) underlying the condition. The neuropathologic features depicted in Fig. 4 are commonly considered to be causative substrates of dementia, or proxies for the disease-driving mechanism(s) [32, 42, 43]. If only < 20% of cases are likely “pure” ADNC, that number may also represent the proportion of dementia cases that are expected to be cured by a therapy aimed at Aβ and tau proteinopathies alone.

The complexity of the dementia-related disease phenotypes may imply that combination therapies will be required. In other words, a single remedy that prevents all amnestic dementia would be wonderful but is not likely to ever be discovered. No biomarker currently exists for some of the common and high-morbidity pathologies (e.g., TDP-43 proteinopathy, which affects up to 40% of persons in advance age [44]), which indicates a considerable problem of a “dirty sample”. As a topical example, even persons that “rule in” to an anti- Aβ clinical trial, due to the presence of Aβ amyloidosis via PET imaging or fluid biomarker studies, may well have a substantial burden of other pathologies. This underscores the need for better biomarkers, including blood based biomarkers for ease of diagnosis and larger sample sizes in clinical trials of dementia to take into account such mixed pathology.

Despite the challenges, there are new opportunities as well. Novel oncology trials are now commonplace to target specific mutations in cancers with, for example, tyrosine kinase inhibitors, checkpoint inhibitors and other modulators of the immune system. This strategy provides potential common ground for therapeutic development relevant to dementia and cancer alike, based on molecular signatures and developing person-specific modulation based on actionable mutations, reflecting that unique molecular changes drive the underlying diseases. This precision medicine paradigm may prove fruitful in the field of aging and dementia, similar to that seen in the field of oncology over the last several decades.

## Conclusions

We can use insights and input from our colleagues in the field of oncology oriented clinical trials to achieve our ultimate goals of leveraging cutting-edge research to achieve translational breakthroughs, while providing at-risk individuals with the bases to make informed and feasible choices, for the benefit of the present and future generations. The successes of future dementia clinical trials probably hinge upon grappling with neurodegenerative diseases’ complexity, and depend on our collective ability to educate patients and clinicians about those complexities, factoring in the risk of relying on clinical strategies that don't modify the disease itself. Thus, a more optimized approach may result in more individuals participating in dementia clinical trials, and more people benefiting from them.

Although the culture of cancer research can provide a helpful model, and give hope for new therapeutic options, there are both challenges and under-appreciated opportunities that make dementia clinical trials unique – from biologic and social standpoints, including various practical and financial issues. Some topical considerations for the contemporary dementia research field (by no means a complete list) include a rational adjustment of the relationships between pharmaceutical companies and academia, rigorous institutional review board oversight, change in restrictive guidelines in clinical trials to be able to study the full population of demented adults, conflict of interest disclosures, the ability to include under-represented minorities in research, and the requirement to disseminate and/or publish trial results even if the study is negative (see Ref [45]). These ideas relate generally to protecting patients from mistakes, mistreatment, and malfeasance—very important considerations! However, there needs to also be a corresponding central focus on protecting the patient from the diseases that afflict them, and providing the patients with better education and data-driven choices.

## Data Availability

No datasets were generated or analysed during the current study.

## Abbreviations

ADNC: Alzheimer’s disease neuropathologic change
LATE-NC: Limbic-predominant age-related TDP-43 encephalopathy neuropathologic change
LBD: Lewy body disease
NCI: National Cancer Institute
SEER: Surveillance, Epidemiology, and End Results
UK-ADRC: University of Kentucky Alzheimer’s Disease Research Center
VCID: Vascular cognitive impairment and dementia
